# Effect of a Brief Web-Based Educational Intervention on Willingness to Consider Human Papillomavirus Vaccination for Children in Japan: Randomized Controlled Trial

**DOI:** 10.2196/28355

**Published:** 2021-09-27

**Authors:** Yukio Suzuki, Akiko Sukegawa, Yutaka Ueda, Masayuki Sekine, Takayuki Enomoto, Etsuko Miyagi

**Affiliations:** 1 Department of Obstetrics and Gynecology Graduate School of Medicine Yokohama City University Yokohama Japan; 2 Division of Gynecologic Oncology, Department of Obstetrics and Gynecology Vagelos College of Physicians and Surgeons Columbia University New York, NY United States; 3 Department of Obstetrics and Gynecology Graduate School of Medicine Osaka University Suita Japan; 4 Department of Obstetrics and Gynecology Graduate School of Medical and Dental Sciences Niigata University Niigata Japan

**Keywords:** human papillomavirus, human papillomavirus vaccination, behavioral insights, behavioral change, web-based randomized controlled trial

## Abstract

**Background:**

The human papillomavirus (HPV) vaccination rate in Japan has fallen to nearly zero since the suspension of governmental proactive recommendations in 2013, owing to the development of purported adverse events.

**Objective:**

This study aimed to evaluate the effects of a brief web-based educational intervention using the theory of behavioral insights on the willingness of adults to consider the HPV vaccine for their daughters and sons.

**Methods:**

We recruited 1660 participants aged 20 years or older in March 2018 via a webpage and provided them with a 10-item questionnaire related to the following aspects: awareness regarding HPV infection and vaccination, willingness for immunization, and actions for prevention. We randomly stratified participants based on sex and age with or without a brief educational intervention involving scientific information presented in an easy-to-read format.

**Results:**

Only 484 (29.2%) of the respondents were aware of the benefits of HPV vaccination. Although only 352 (21.2%) of the respondents displayed a willingness for immunization of their daughters, there were 40 (4.8%) more respondents in the intervention group with this willingness (adjusted odds ratio [aOR] 1.32, 95% CI 1.04-1.69). In a subanalysis, the willingness toward vaccination for daughters in men was significantly higher in the intervention group (aOR 1.46, 95% CI 1.05-2.02). However, such a difference was not observed among women (aOR 1.20, 95% CI 0.83-1.73).

**Conclusions:**

This study suggests that a brief web-based educational intervention increases the willingness of adults to consider the HPV vaccine for their children, especially among men. Thus, providing adequate information to men may be a useful strategy to improve the currently low rates of HPV vaccination.

**Trial Registration:**

UMIN Clinical Trials Registry UMIN000049745 (UMIN-CTR); https://upload.umin.ac.jp/cgi-open-bin/ctr_e/ctr_view.cgi?recptno=R000049745

## Introduction

### Background

In Japan, the human papillomavirus (HPV) nonavalent vaccine was approved by the Pharmaceuticals and Medical Devices Agency (PMDA) for girls aged 9 years or older in July 2020 [1]. The corresponding quadrivalent inoculation was ratified for boys in the same age group in December 2020 [1]. Nevertheless, the level of awareness regarding HPV, the resultant cancer (cervical cancer [CC]), and the vaccine needs to be higher in the Japanese population [2,3]. Although vaccination is the exclusive means of preventing HPV infection, the immunization rate has fallen below 1% owing to subsequent adverse events [4], which are regarded as functional disorders. These cases were reported repeatedly in Japanese media in sensational ways [2,4]. As a result of the dissemination of misinformation and the misunderstanding of the HPV vaccine, most Japanese people have distrust toward the HPV vaccine [2]. Thus, the immunization rate of the bivalent or quadrivalent HPV vaccine for the target population from the 6th grade of elementary school students to the 1st grade of high school students was 0.8% in 2018 [5]. This has persisted from 2013 for almost 8 years [4-6].

According to the strategy of the World Health Organization devised in 2019 for controlling CC [7], by 2030, 90% of girls worldwide would be vaccinated with the HPV vaccine by the age of 15 years. Malaysia, Mexico, Bhutan, Brunei, and Rwanda have achieved an immunization rate of 90% or higher in the target population [7]. Furthermore, in Australia, the success of two national programs, the National Cervical Screening Program (NCSP) and the Australian National HPV Vaccination Program (NHVP), resulted in achievement of the threshold for rare cancer in 2020 [8]. By 2028, the estimated number of cases would be less than 4 per 100,000 [9], which is the threshold for elimination [8].

In addition to HPV-related cancers, vulvar, anal, and throat malignancies may be prevented by the vaccine [9,10]. A Japanese research group provided evidence of a significant reduction in the incidence of cervical intraepithelial neoplasia grade 3 or higher in vaccinated women [11].

Specific information regarding the effects and adverse events of HPV vaccines needs to be fully disseminated to the Japanese population. Considering this, approximately 40% of the population is willing to be vaccinated [2]. Therefore, it is critical to disseminate adequate scientific knowledge regarding the beneficial effects of the vaccine so that people in the target age group actually take the vaccine.

### Goal of the Study

Vaccine awareness programs are necessary, and campaigns through the media and social network services can play significant roles [2,4]. In an information-overloaded society, people frequently make decisions related to health issues based on a bunch of information [12]. Therefore, it is critical to consider the influence of behavioral insights to promote change [13]. This broadly refers to concrete approaches based on the knowledge of behavioral science and economics. The Easy, Attractive, Social, and Timely (EAST) principles are a simple way of applying behavioral insights to interventions and have been used to change human awareness and behavior [13]. This study aimed to assess the effects of these behavioral insights utilizing brief scientific information on vaccine benefits, along with statistics on CC.

## Methods

### Study Design and Participants

**Figure 1:**
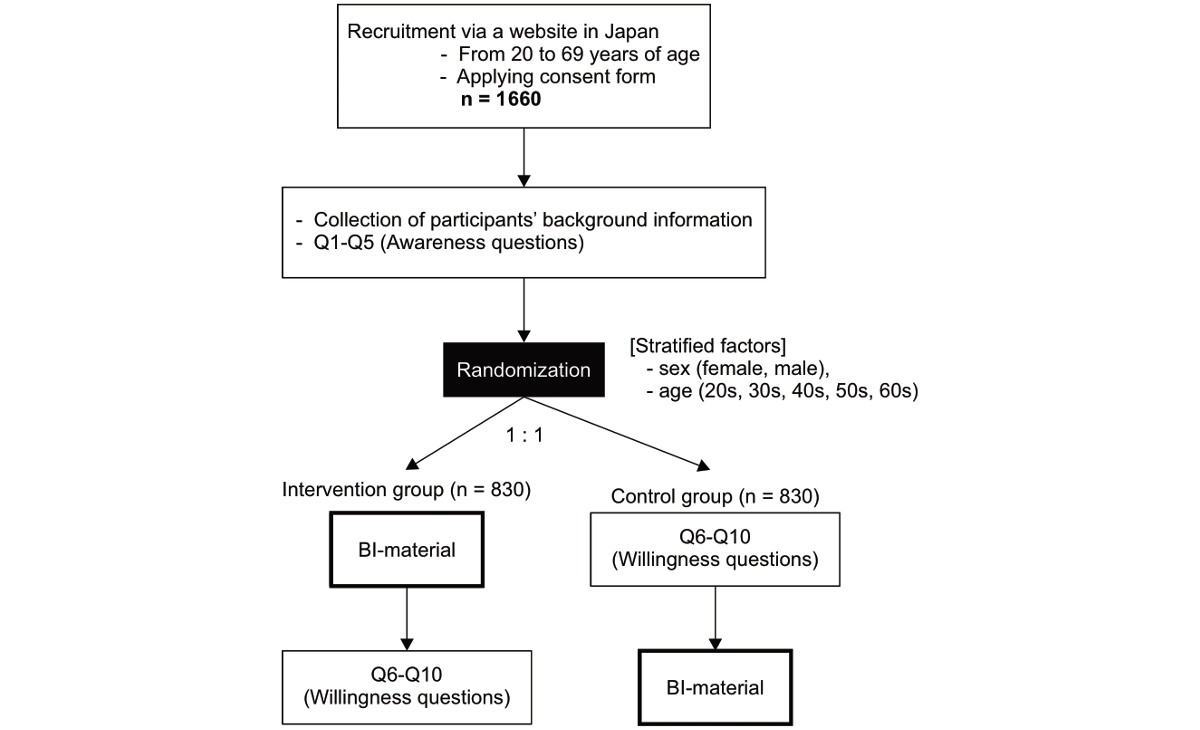
Flow diagram of the randomization. BI-material: behavioral insights material featuring brief scientific information presented in an easy-to-read format.

**Figure 2:**
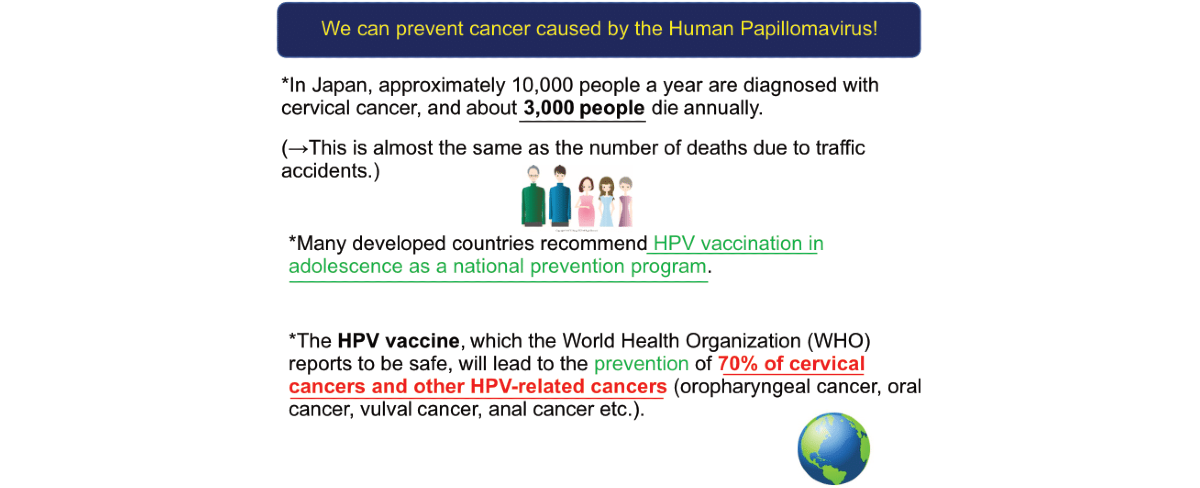
Behavioral insights material.

We recruited a total of 1660 participants in March 2018 via a specially designed webpage for this study. These were registered members of the research panel owned by Macromill Inc (Tokyo, Japan). The participants were 20 years old or above as on March 12-13, 2018. They were recruited until the target sample size was fulfilled. We randomly assigned each participant to respond to an identical questionnaire after (intervention group) or prior to (control group) providing behavioral insights material (BI-material) featuring brief scientific information presented in an easy-to-read format (as displayed in Figures 1 and 2).

### Informational Material and Behavioral Insights

Informational materials are designed primarily to increase effectiveness; they are based on a specific purpose rather than a template. In this study, first, we provided one-page BI-material containing three sentences, which were deemed to be in line with scientific evidence and objective facts associated with the theory of behavioral change and economics. This theory was suggested and represented by Richard Thaler [14]. The first sentence of our material reflected the Japanese epidemiological data on CC (Figure 2), which was intended for the framing effect [15,16]. For example, a statement like “Cervical cancer is the 11th most frequent malignancy among women in Japan” or “2.8% of women diagnosed with cervical cancer die” does not emphasize the deterioration in health. Therefore, this would not contribute to general behavior change. Additionally, we applied the prospect theory by drawing similarities between the number of deaths due to CC in Japan and fatalities due to traffic accidents. Second, to promote a change in the consciousness of participants, the presentation of social norms was included as part of the EAST framework (S-social) in the second sentence of the BI-material (Figure 2). This was employed for devising behavioral insights of greater effectiveness [17]. Third, heuristics (awareness of prejudice and intuition) [18] hypothesized a reduced interest in male participants in case the HPV immunization focused exclusively on CC. Therefore, it was conveyed that non-CC HPV malignancies are preventable through HPV vaccination [9]. The first author (YS) is one of the specialists of the behavioral design team in Yokohama, which was established as the first nongovernmental nudge unit in Japan [19]. Thus, this BI-material was created given the behavioral insights methodology.

### Randomization

Participants were randomly allocated (1:1) to each group. The intervention was performed using an automatic web-based allocation system stratified by the sex (female/women and male/men) and age (20s, 30s, 40s, 50s, and 60s) of the participants. Randomization was performed by the web-research system of Macromill Inc. Participants and investigators were blinded to the distribution (double-blinded). Once the upper limit of each stratum was reached, new participants could not be added to the web system. This ensured uniform distribution of the stratification factors. In the intervention group, we provided the BI-material prior to answering questions related to preventive awareness, following consent for the online study. The control group was provided with the same material as that provided to the intervention group after all responses were completed.

### Questionnaire

The participant demographics included age, sex, marital status, number of children, sexual experience, household income, medical background, education, and tobacco use. For female participants, we also collected information regarding HPV vaccination history and previous pap screening. Medical background was defined as follows: a respondent who had a history of working as a medical professional (medical doctor, dentist, nurse, or allied health care professional) or who attended medical school.

The respondents completed a 10-item questionnaire. The first half determined HPV awareness as background information, and the second half addressed willingness to receive HPV vaccines and undergo screening tests. The respondents were instructed to answer “Yes” or “No” for each question.

The awareness questions were as follows:

Q1. It is possible to detect both cancer and precancerous lesions through CC screening.

Q2. Sexual experience is associated with HPV infection.

Q3. CC screening is necessary for women even after vaccination.

Q4. I have heard of the benefits of the HPV vaccine.

Q5. I have heard of the adverse events associated with the HPV vaccine.

The willingness questions were as follows:

Q6. If you have/had a daughter, do/would you consider getting her vaccinated against HPV?

Q7. If you have/had a son, do/would you consider getting him vaccinated against HPV?

Q8. Would you consider undergoing a pap smear? If male, will you want your family or partner to undergo a smear test?

Q9. Would you undergo the cancer screening tests recommended by the government?

Q10. Do you plan informing family members, friends, or others about cancer prevention and screening (through Facebook, LINE, Twitter, etc)?

### Statistical Analysis

#### Tests Performed

Statistical evaluation comprised the Student *t* test, the chi-square (*χ*^2^) test, and multiple regression analyses. These were performed using SPSS, version 27 (IBM Corp).

#### Power and Statistical Significance

The sample size was calculated as 80% powered to detect a 10% effect in the intervention group (increased from 40% in the control group to 50% in the intervention group) with a two-sided *P* value of .05. *P* values less than .05 were regarded as significant. The hypothetical baseline willingness rate in the control group was determined based on our previous study [2]. The sample size was calculated as 776 when the effect of the intervention estimated a 10% increase. The number of participants recruited was double of the calculated sample size because of the difficulty in estimating the baseline willingness and the intervention effect.

#### Odds Ratio

The adjusted odds ratios (aORs) related to the background knowledge level from the binominal logistic regression were analyzed to assess differences in both groups. In the subanalysis, questions Q2 and Q4, for which the responses showed a significant difference in the rate of awareness between the two groups (data not shown), were included as covariates in the aOR in the analysis for men. Questions Q1, Q2, and Q5, for which the responses showed a significant difference in the rate of awareness between the two groups (data not shown), were included as covariates in the aOR in the analysis for women.

### Ethical Approval and Funding

The study protocol was approved by the Institutional Research Ethics Committee of Yokohama City University School of Medicine (A180200004). The trial registration number is UMIN000049745. We received research funding from the Japan Agency for Medical Research and Development (grant number 15ck0106103h0102). The survey was outsourced to Macromill Inc.

## Results

### Analysis of Participant Demographics

From March 12 to 13, 2018, 1660 participants were recruited. Stratifying factors, such as sex and age, were evenly allocated. The following variables displayed no significant differences between the intervention and control groups: marital status (*P*=.96), children (*P*=.84), household income (*P*=.58), sexual experience (*P*=.26), education (*P*=.44), medical background (*P*=.50), and tobacco use (*P*=.64) (Table 1).

**Table 1:** Characteristics and knowledge level of the participants recruited.

Characteristic		All (N=1660)	Intervention group (n=830)	Control group (n=830)	*P* value^a^
**Sex, n (%)**					>.99
	Male	830 (50.0)	415 (50.0)	415 (50.0)	
	Female	830 (50.0)	415 (50.0)	415 (50.0)	
Age (years), mean (SD)		44.7 (14.0)	44.6 (14.1)	44.8 (14.0)	.77
Age range (years)		20-69	20-69	20-69	
**Age groups (years), n (%)**					>.99
	20-29	332 (20.0)	166 (20.0)	166 (20.0)	
	30-39	332 (20.0)	166 (20.0)	166 (20.0)	
	40-49	332 (20.0)	166 (20.0)	166 (20.0)	
	50-59	332 (20.0)	166 (20.0)	166 (20.0)	
	≥60	332 (20.0)	166 (20.0)	166 (20.0)	
**Marital status, n (%)**					.96
	Married	1013 (61.0)	507 (61.1)	506 (61.0)	
	Unmarried	647 (39.0)	323 (38.9)	324 (39.0)	
**Children, n (%)**					.84
	Yes	904 (54.5)	454 (54.7)	450 (54.2)	
	No	756 (45.5)	376 (45.3)	380 (45.8)	
**Sexual experience, n (%)**					.26
	Experienced	1298 (78.2)	659 (79.4)	639 (77.0)	
	Not experienced	145 (8.7)	64 (7.7)	81 (9.8)	
	Declined to answer	217 (13.1)	107 (12.9)	110 (13.3)	
**Household income (million yen/year)^b^, n (%)**					.58
	<2	120 (7.2)	61 (7.3)	59 (7.1)	
	2-4	316 (19.0)	147 (17.7)	169 (20.4)	
	4-6	384 (23.1)	199 (24.0)	185 (22.3)	
	6-8	208 (12.5)	105 (12.7)	103 (12.4)	
	8-10	153 (9.2)	80 (9.6)	73 (8.8)	
	>10	126 (7.7)	60 (7.2)	66 (8.0)	
	Declined to answer	353 (21.2)	178 (21.4)	175 (21.1)	
**Medical background, n (%)**					.50
	Medical	116 (7.0)	54 (6.5)	62 (7.5)	
	Nonmedical	1544 (93.0)	776 (93.5)	769 (92.5)	
**Education, n (%)**					.44
	Less than high school graduate	34 (2.0)	17 (2.0)	17 (2.0)	
	High school graduate	467 (28.1)	237 (28.6)	230 (27.7)	
	More than high school graduate	1159 (69.8)	576 (69.4)	583 (70.2)	
**Tobacco use, n (%)**					.64
	Smoker	312 (18.8)	162 (19.5)	150 (18.1)	
	Nonsmoker	896 (54.0)	439 (52.9)	457 (55.1)	
	Previous smoker	452 (27.2)	229 (27.6)	223 (26.9)	
**Q1. It is possible to detect both cancer and precancerous lesions through cervical cancer (CC) screening, n (%)**					.11
	Already known	670 (40.4)	319 (38.4)	351 (42.3)	
	Not known	990 (59.6)	511 (61.6)	479 (57.7)	
**Q2. Sexual experience is associated with HPV^c^ infection, n (%)**					.001
	Already known	911 (54.9)	423 (51.0)	488 (58.8)	
	Not known	749 (45.1)	407 (49.0)	342 (41.2)	
**Q3. Cervical cancer screening is necessary for women even after vaccination, n (%)**					.06
	Already known	518 (31.2)	277 (33.4)	241 (29.0)	
	Not known	1142 (68.8)	553 (66.6)	589 (71.0)	
**Q4. Benefits of HPV vaccination, n (%)**					.004
	Already known	484 (29.2)	269 (32.4)	215 (25.9)	
	Not known	1176 (70.8)	561 (67.6)	615 (74.1)	
**Q5. Adverse events associated with HPV vaccination, n (%)**					.004
	Already known	543 (32.7)	299 (36.0)	244 (29.4)	
	Not known	1117 (67.3)	531 (64.0)	586 (70.6)	
**Last pap^d^ screening^e^, n (%)**					.87
	<2 years	341 (41.1)	178 (42.9)	163 (39.3)	
	2-5 years	107 (12.9)	54 (13.0)	53 (12.8)	
	>5 years	125 (15.1)	57 (13.7)	68 (16.4)	
	Never	232 (28.0)	113 (27.2)	119 (28.7)	
	Unknown	25 (3.0)	13 (3.1)	12 (2.9)	
**HPV vaccination^e^, n (%)**					.70
	Already vaccinated	36 (4.3)	21 (5.1)	15 (3.6)	
	Not yet vaccinated	595 (71.7)	303 (73.0)	292 (70.4)	
	Unknown	199 (24.0)	91 (21.9)	108 (26.0)	

^a^*P* values are estimated using the chi-square and Student *t* tests.

^b^1 USD=110 JPY.

^c^HPV: human papillomavirus.

^d^Pap: Papanicolaou test.

^e^Only female participants aged 20 years or older (n=830).

### HPV Awareness Analysis

For questions Q1 to Q5 regarding HPV awareness, the recognition rate for Q2 was significantly higher (by 7.8%) in the control group than in the intervention group. For questions Q4 and Q5, the values in the intervention group were significantly higher (by 6.5% and 6.6%, respectively) than those in the control group (Table 1).

Only 484 (29.2%) of the respondents were aware of the benefits of HPV immunization (Q4), whereas 543 (32.7%) were aware of the adverse effects (Q5).

### Willingness to Consider Children’s Vaccination

Only 352 (21.2%) of the respondents displayed a favorable attitude toward HPV immunization for their daughters (Q6). However, an additional 40 (4.8%) participants responded affirmatively in the intervention group (aOR 1.32, 95% CI 1.04-1.69) compared to those in the control group (Table 2). For Q7, there were additional 33 (3.9%) satisfied respondents willing to consider vaccination for their sons in the intervention group (aOR 1.38, 95% CI 1.05-1.80) compared to those in the control group (Table 2).

The number of respondents expressing a desire to receive a screening test (Q8) (*P*=.37) or to communicate HPV-related issues (Q10) did not increase (*P*=.17) (Table 2).

**Table 2:** Comparison of attitudes toward human papillomavirus vaccination and screening tests according to intervention.

Variable		All (N=1660), n (%)	Intervention (n=830), n (%)	Control (n=830), n (%)	Yes response vs other			
					OR^a^ (95% CI)	*P* value^b^	Adjusted OR^c^ (95% CI)	*P* value^b^
**Q6. If you have/had a daughter, do/would you consider getting her vaccinated against HPV^d^?**								
	Yes	352 (21.2)	196 (23.6)	156 (18.8)	1.34 (1.05-1.69)	.02	1.32 (1.04-1.69)	.02
	No	240 (14.5)	115 (13.9)	125 (15.1)				
	I’m not sure	1068 (64.3)	519 (62.5)	549 (66.1)				
**Q7. If you have/had a son, do/would you consider getting him vaccinated against HPV?**								
	Yes	273 (16.4)	153 (18.4)	120 (14.5)	1.34 (1.03-1.74)	.03	1.38 (1.05-1.80)	.02
	No	254 (15.3)	127 (15.3)	127 (15.3)				
	I’m not sure	1133 (68.3)	550 (66.3)	583 (70.2)				
**Q8. Would you consider undergoing a pap^e^ smear? If male, will you want your family or partner to have a smear?**								
	Yes	1186 (71.4)	601 (72.4)	585 (70.5)	1.10 (0.89-1.36)	.39	1.10 (0.89-1.37)	.37
	No	474 (28.6)	229 (27.6)	245 (29.5)				
**Q9. Would you undergo the cancer screening tests recommended by the government?**								
	Yes	1128 (68.0)	574 (69.2)	554 (66.7)	1.12 (0.91-1.37)	.29	1.12 (0.91-1.38)	.30
	No	532 (32.0)	256 (30.8)	276 (33.3)				
**Q10. Do you plan informing family members, friends, or others about cancer prevention and screening (through Facebook, LINE, Twitter, etc)?**								
	Yes	760 (45.8)	396 (47.7)	364 (43.9)	1.17 (0.96-1.42)	.12	1.15 (0.94-1.40)	.17
	No	900 (54.2)	434 (52.3)	466 (56.1)				

^a^OR: odds ratio.

^b^*P* value estimated using binomial logistic regression analysis.

^c^Q2, Q4, and Q5 were included as covariates in the adjusted OR.

^d^HPV: human papillomavirus.

^e^Pap: Papanicolaou test.

### Sex-Wise Attitudes Toward HPV Vaccination

Table 3 presents the subanalysis results according to sex.

Differences were identified in Q6 (men: aOR 1.46, 95% CI 1.05-2.02 vs women: aOR 1.20, 95% CI 0.83-1.73) and Q7 (men: aOR 1.53, 95% CI 1.08-2.18 vs women: aOR 1.21, 95% CI 0.80-1.83). The willingness to consider vaccination for daughters in men was significantly higher in the intervention group (by 8.2%, *P*=.02; Multimedia Appendix 1); however, such a difference was not observed in women (*P*=.22; Table 3, Multimedia Appendix 2).

In an overall comparison between men and women irrespective of intervention, the willingness to consider vaccination for daughters in men was significantly higher than that in women (25.1% vs 17.3%, *P*<.001), and the willingness to consider vaccination for sons was also higher in men than in women (20.1% vs 12.8%, *P*<.001).

In the intervention group, higher rates were identified in men than in women for Q6 (29.2% vs 18.1%, *P*<.001) and Q7 (23.9% vs 13.0%, *P*<.001). While in the control group, differences were not identified between men and women for Q6 (21.0% vs 16.6%, *P*=.11) and Q7 (16.4% vs 12.5%, *P*=.11).

**Table 3:** Comparison of attitudes toward human papillomavirus vaccination and screening tests according to sex.

Variable			Yes response vs other						
			OR^a^ (95% CI)		*P* value^b^		Adjusted OR^c^ (95% CI)		*P* value^b^
**Q6. If you have/had a daughter, do/would you consider getting her vaccinated against HPV^d^?**									
	Men	1.55 (1.13-2.13)		.01		1.46 (1.05-2.02)		.03	
	Women	1.11 (0.77-1.59)		.58		1.20 (0.83-1.73)		.33	
**Q7. If you have/had a son, do/would you consider getting him vaccinated against HPV?**									
	Men	1.60 (1.13-2.26)		.01		1.53 (1.08-2.18)		.02	
	Women	1.04 (0.70-1.57)		.84		1.21 (0.80-1.83)		.38	
**Q8. Would you consider undergoing a pap^e^ smear? If male, will you want your family or partner to have a smear?**									
	Men	1.05 (0.78-1.42)		.76		1.04 (0.77-1.42)		.78	
	Women	1.15 (0.85-1.56)		.36		1.18 (0.87-1.61)		.30	
**Q9. Would you undergo the cancer screening tests recommended by the government?**									
	Men	1.01 (0.76-1.34)		.94		1.02 (0.76-1.36)		.92	
	Women	1.25 (0.93-1.69)		.15		1.31 (0.96-1.79)		.09	
**Q10. Do you plan informing family members, friends, or others about cancer prevention and screening (through Facebook, LINE, Twitter, etc)?**									
	Men	1.27 (0.96-1.67)		.09		1.24 (0.94-1.64)		.13	
	Women	1.08 (0.82-1.42)		.58		1.11 (0.84-1.47)		.47	

^a^OR: odds ratio.

^b^*P* value estimated using binomial logistic regression analysis.

^c^Q2 and Q4 were included as covariates in the adjusted OR in the analysis involving men. Q1, Q2, and Q5 were included as covariates in the adjusted OR in the analysis involving women.

^d^HPV: human papillomavirus.

^e^Pap: Papanicolaou test.

## Discussion

### Principal Findings

We conducted a web-based randomized controlled trial (RCT) to assess the benefits of BI-material employing brief scientific information and its ability to motivate individuals to consider the HPV vaccine for their children. Our results showed that providing brief scientific information could increase the willingness to consider HPV vaccination for daughters and sons. This effect was observed typically among male participants. Similar minor interventions may potentially modify mindsets favorably. However, such brief digital information failed to affect the mindset in women. A possible reason why the intervention was more effective among men than women is that women had a more negative image toward HPV vaccination. There was a significant difference between men and women in the awareness level. Overall, 45.7% of women responded that they know about the adverse events of HPV vaccination, while this rate was 19.8% in men (data not shown). Such awareness might have influenced the difference in the intervention effect.

In terms of the COVID-19 pandemic, a difference in awareness of prevention strategies was observed between men and women [20]; therefore, it is essential to build a method appropriate for sex subgroups to transform general behavior via the internet and social networking services.

### Comparison With Prior Work

According to a systematic review regarding the effect of the pedagogical approach on social awareness or action, more reliable and validated studies are required to change the perception or mindset of the target population [21]. This paves the way for mitigating hesitancy toward vaccination [21]. Therefore, this RCT may be valuable to ensure a change in public attitudes toward vaccination.

A systematic review revealed that general communication about childhood vaccination resulted in a positive change by 20%; however, this excluded the HPV vaccine [21]. In contrast, the brief educational intervention was observed to improve the willingness to consider the HPV vaccine for daughters and sons, with aORs of 1.32 and 1.38, respectively (Table 2). Therefore, when compared to the review data of the in-person approach [21], the aORs were not small. Furthermore, the BI-material was designed by a specialist of behavioral insight, resulting in a moderate cost; therefore, this intervention is more reliable and cost-effective than those used in previous studies.

A US RCT reported that education through social media is effective for improving general awareness regarding vaccination. However, the study was limited to a sample of 58 participants [22]. A phase 2 Japanese trial evaluated how the extent of intervention affected HPV vaccination acceptance and reported that providing appropriate medical information resulted in beneficial effects [23]. Thus, this study proved the utility of effective and adequate guidance in improving vaccine acceptance. There is a paucity of literature on the efficacy of pragmatic educational materials for HPV vaccination promotion [22]. Therefore, more extensive trials focusing on the mode of endorsing HPV vaccination like our trial are required for evidence-based promotion.

### Limitations

We recognize several limitations in this study. First, the sustainability of effective change was not evaluated. Typically, with respect to health issues, taking action requires time. Therefore, a study should assess not only a change in mindset, but also the appropriate course of action. We have already performed an RCT (UMIN000039273) assessing the sustainability of general acceptance and concrete behavior for HPV vaccination. Second, the impact of the study on the behavioral outcome was unclear; specific vaccination functioning needs to be tracked. Third, selection bias was present as the respondents were Japanese individuals enrolled by an internet survey company.

Based on the sex-wise subanalysis, improved information in male participants may be the key to improve the rate of HPV vaccination in Japan. Additionally, video-based content with patient feedback is expected to result in a broader impact. Thus, a varied approach for men and women may be required.

### Conclusions

#### Inference of the Study Findings

Our study revealed a positive outlook toward HPV vaccination following a brief web-based educational intervention, especially among men. Such an approach is extremely effective to overcome challenges related to communication and information overload.

#### Impact of the Findings

A brief web-based educational intervention based on the theory of behavioral insights increases the willingness of Japanese adults to consider the HPV vaccine for their daughters and sons.

## Appendix

Multimedia Appendix 1 Comparison of attitudes toward human papillomavirus vaccination and screening tests in men.

Multimedia Appendix 2 Comparison of attitudes toward human papillomavirus vaccination and screening tests in women.

Multimedia Appendix 3 CONSORT-eHEALTH checklist (V 1.6.2).

## Abbreviations

aOR: adjusted odds ratio
BI: behavioral insights
CC: cervical cancer
EAST: Easy, Attractive, Social, and Timely
HPV: human papillomavirus
OR: odds ratio
RCT: randomized controlled trial

## References

[1] (2020). New drugs approved in May 2020. Pharmaceuticals and Medical Devices Agency.

[2] Suzuki Y, Sukegawa A, Nishikawa A, Kubota K, Motoki Y, Asai-Sato M, Ueda Y, Sekine M, Enomoto T, Hirahara F, Yamanaka T, Miyagi E (2019). Current knowledge of and attitudes toward human papillomavirus-related disease prevention among Japanese: A large-scale questionnaire study. J Obstet Gynaecol Res.

[3] Hanley SJB, Yoshioka E, Ito Y, Konno R, Sasaki Y, Kishi R, Sakuragi N (2014). An exploratory study of Japanese fathers' knowledge of and attitudes towards HPV and HPV vaccination: does marital status matter?. Asian Pac J Cancer Prev.

[4] Sekine M, Kudo R, Yamaguchi M, Hanley SJB, Hara M, Adachi S, Ueda Y, Miyagi E, Ikeda S, Yagi A, Enomoto T (2020). Japan's Ongoing Crisis on HPV Vaccination. Vaccines (Basel).

[5] Ministry of Health, Labour and Welfare Committee on vaccine adverse reactions. Ministry of Health, Labour and Welfare.

[6] Hanley SJB, Yoshioka E, Ito Y, Kishi R (2015). HPV vaccination crisis in Japan. The Lancet.

[7] (2019). Global strategy to accelerate the elimination of cervical cancer as a public health problem. World Health Organization.

[8] Hall MT, Simms KT, Lew J, Smith MA, Brotherton JM, Saville M, Frazer IH, Canfell K (2019). The projected timeframe until cervical cancer elimination in Australia: a modelling study. The Lancet Public Health.

[9] Luostarinen T, Apter D, Dillner J, Eriksson T, Harjula K, Natunen K, Paavonen J, Pukkala E, Lehtinen M (2018). Vaccination protects against invasive HPV-associated cancers. Int J Cancer.

[10] Lei J, Ploner A, Elfström KM, Wang J, Roth A, Fang F, Sundström K, Dillner J, Sparén P (2020). HPV Vaccination and the Risk of Invasive Cervical Cancer. N Engl J Med.

[11] Kudo R, Yamaguchi M, Sekine M, Adachi S, Ueda Y, Miyagi E, Hara M, Hanley SJB, Enomoto T (2019). Bivalent Human Papillomavirus Vaccine Effectiveness in a Japanese Population: High Vaccine-Type-Specific Effectiveness and Evidence of Cross-Protection. J Infect Dis.

[12] Soroya SH, Farooq A, Mahmood K, Isoaho J, Zara S (2021). From information seeking to information avoidance: Understanding the health information behavior during a global health crisis. Inf Process Manag.

[13] Behavioural insights. Organisation for Economic Co-operation and Development.

[14] Thaler R, Sunstein C (2009). Nudge: Improving Decisions About Health, Wealth, and Happiness.

[15] Kahneman D, Tversky A (1979). Prospect Theory: An Analysis of Decision under Risk. Econometrica.

[16] Gong J, Zhang Y, Yang Z, Huang Y, Feng J, Zhang W (2013). The framing effect in medical decision-making: a review of the literature. Psychol Health Med.

[17] EAST: Four Simple Ways to Apply Behavioural Insights. Behavioural Insights Team.

[18] Coussens S (2018). Behaving Discretely: Heuristic Thinking in the Emergency Department. SSRN Journal.

[19] Yokohama Behavioral Insights and Design Team (YBiT).

[20] Li S, Feng B, Liao W, Pan W (2020). Internet Use, Risk Awareness, and Demographic Characteristics Associated With Engagement in Preventive Behaviors and Testing: Cross-Sectional Survey on COVID-19 in the United States. J Med Internet Res.

[21] Kaufman J, Ryan R, Walsh L, Horey D, Leask J, Robinson P, Hill S (2018). Face-to-face interventions for informing or educating parents about early childhood vaccination. Cochrane Database Syst Rev.

[22] Brandt HM, Sundstrom B, Monroe CM, Turner-McGrievy G, Larsen C, Stansbury M, Magradey K, Gibson A, West DS (2020). Evaluating a Technology-Mediated HPV Vaccination Awareness Intervention: A Controlled, Quasi-Experimental, Mixed Methods Study. Vaccines (Basel).

[23] Mizumachi K, Aoki H, Kitano T, Onishi T, Takeyama M, Shima M (2021). How to recover lost vaccine acceptance? A multi-center survey on HPV vaccine acceptance in Japan. J Infect Chemother.

