# Supplementary material for: Effect of a Brief Web-Based Educational Intervention on Willingness to Consider Human Papillomavirus Vaccination for Children in Japan: Randomized Controlled Trial
Source: J Med Internet Res. 2021 Sep 27;23(9):e28355. doi: 10.2196/28355 (PMC8506261; doi:10.2196/28355)
Supplement: Multimedia Appendix 1 [file jmir_v23i9e28355_app1.docx]

**Multimedia Appendix 1.** Comparison of attitudes toward human papillomavirus vaccination and screening tests in men.

|  |  | All | Intervention | Control | Yes vs. Other | | | |  |
| --- | --- | --- | --- | --- | --- | --- | --- | --- | --- |
|  |  | *n* (%) | *n* (%) | *n* (%) | OR^b^ (95% CI^c^) | *P* | Adjusted OR (95% CI) | *P* |  |
|  |  |  |  |  |  |  |  |  |  |
| Q6 |  |  |  |  |  |  |  |  |  |
|  | Yes | 208 (25.1) | 121 (29.2) | 87 (21.0) | 1.55 (1.13-2.13) | *.01* | 1.46 (1.05-2.02) | *.03* |  |
|  | No | 81 (9.8) | 37 (8.9) | 44 (10.6) |  |  |  |  |  |
|  | I’m not sure | 541 (65.2) | 257 (61.9) | 284 (68.4) |  |  |  |  |  |
| Q7 |  |  |  |  |  |  |  |  |  |
|  | Yes | 167 (20.1) | 99 (23.9) | 68 (16.4) | 1.60 (1.13-2.26) | *.01* | 1.53 (1.08-2.18) | *.02* |  |
|  | No | 101 (12.2) | 49 (11.8) | 52 (12.5) |  |  |  |  |  |
|  | I’m not sure | 562 (67.7) | 267 (64.3) | 295 (71.1) |  |  |  |  |  |
| Q8 |  |  |  |  |  |  |  |  |  |
|  | Yes | 590 (71.1) | 297 (71.6) | 293 (70.6) | 1.05 (0.78-1.42) | *.76* | 1.04 (0.77-1.42) | *.78* |  |
|  | No | 240 (28.9) | 118 (28.4) | 122 (29.4) |  |  |  |  |  |
| Q9 |  |  |  |  |  |  |  |  |  |
|  | Yes | 539 (64.9) | 270 (65.1) | 269 (64.8) | 1.01 (0.76-1.34) | *.94* | 1.02 (0.76-1.36) | *.92* |  |
|  | No | 291 (35.1) | 145 (34.9) | 146 (35.2) |  |  |  |  |  |
| Q10 |  |  |  |  |  |  |  |  |  |
|  | Yes | 356 (42.9) | 190 (45.8) | 166 (40.0) | 1.27 (0.96-1.67) | *.09* | 1.24 (0.94-1.64) | *.13* |  |
|  | No | 474 (57.1) | 225 (54.2) | 249 (60.0) |  |  |  |  |  |
| ^a^HPV, Human papillomavirus;  ^b^OR, Odds ratio;  ^c^CI, Confidence interval | | | | |  |  |  |  |  |
| *P* value estimated using binomial logistic regression analysis. | | | | | | | |  |  |
| Q2 and Q4 were included as covariates in the adjusted OR. | | | | | | | | | |
| Q6 If you have/had a daughter, do/would you consider getting her vaccinated against HPV? | | | | | | | | | |
| Q7 If you have/had a son, do/would you consider getting him vaccinated against HPV? | | | | | | | | | |
| Q8 Would you consider undergoing a pap smear? If male, will you regard your family or partner to have a smear? | | | | | | | | | |
| Q9 Would you undergo the cancer screening tests recommended by the government? | | | | | | | | | |
| Q10 Do you plan informing family members, friends, or others about cancer prevention and screening (through Facebook, LINE, Twitter, etc.)? | | | | | | | | | |
