# Supplementary material for: Effect of a Brief Web-Based Educational Intervention on Willingness to Consider Human Papillomavirus Vaccination for Children in Japan: Randomized Controlled Trial
Source: J Med Internet Res. 2021 Sep 27;23(9):e28355. doi: 10.2196/28355 (PMC8506261; doi:10.2196/28355)
Supplement: Multimedia Appendix 2 [file jmir_v23i9e28355_app2.docx]

**Multimedia Appendix 2.** Comparison of attitudes toward human papillomavirus vaccination and screening tests in women.

|  |  | All | Intervention | Control | Yes vs. Other | | | |  |
| --- | --- | --- | --- | --- | --- | --- | --- | --- | --- |
|  |  | *n* (%) | *n* (%) | *n* (%) | OR^b^ (95% CI^c^) | *P* | Adjusted OR (95% CI) | *P* |  |
| Q6 |  |  |  |  |  |  |  |  |  |
|  | Yes | 144 (17.3) | 75 (18.1) | 69 (16.6) | 1.11 (0.77-1.59) | *.58* | 1.20 (0.83-1.73) | *.33* |  |
|  | No | 159 (19.2) | 78 (18.8) | 81 (19.5) |  |  |  |  |  |
|  | I’m not sure | 527 (63.5) | 262 (63.1) | 265 (63.9) |  |  |  |  |  |
| Q7 |  |  |  |  |  |  |  |  |  |
|  | Yes | 106 (12.8) | 54 (13.0) | 52 (12.5) | 1.04 (0.70-1.57) | *.84* | 1.21 (0.80-1.83) | *.38* |  |
|  | No | 153 (18.4) | 78 (18.8) | 75 (18.1) |  |  |  |  |  |
|  | I’m not sure | 571 (68.8) | 283 (68.2) | 288 (69.4) |  |  |  |  |  |
| Q8 |  |  |  |  |  |  |  |  |  |
|  | Yes | 596 (71.8) | 304 (73.3) | 292 (70.4) | 1.15 (0.85-1.56) | *.36* | 1.18 (0.87-1.61) | *.30* |  |
|  | No | 234 (28.2) | 111 (26.7) | 123 (29.6) |  |  |  |  |  |
| Q9 |  |  |  |  |  |  |  |  |  |
|  | Yes | 589 (71.0) | 304 (73.3) | 285 (68.7) | 1.25 (0.93-1.69) | *.15* | 1.31 (0.96-1.79) | *.09* |  |
|  | No | 241 (29.0) | 111 (26.7) | 130 (31.3) |  |  |  |  |  |
| Q10 |  |  |  |  |  |  |  |  |  |
|  | Yes | 404 (48.7) | 206 (49.6) | 198 (47.7) | 1.08 (0.82-1.42) | *.58* | 1.11 (0.84-1.47) | *.47* |  |
|  | No | 426 (51.3) | 209 (50.4) | 217 (52.3) |  |  |  |  |  |
| ^a^HPV, Human papillomavirus;  ^b^OR, Odds ratio;  ^c^CI, Confidence interval | | | | |  |  |  |  |  |
| *P* value estimated using binomial logistic regression analysis. | | | | | | | |  |  |
| Q1, Q2, and Q5 were included as covariates in the adjusted OR. | | | | | | | | | |
| Q6 If you have/had a daughter, do/would you consider getting her vaccinated against HPV? | | | | | | | | | |
| Q7 If you have/had a son, do/would you consider getting him vaccinated against HPV? | | | | | | | | | |
| Q8 Would you consider undergoing a pap smear? If male, will you regard your family or partner to have a smear? | | | | | | | | | |
| Q9 Would you undergo the cancer screening tests recommended by the government? | | | | | | | | | |
| Q10 Do you plan informing family members, friends, or others about cancer prevention and screening (through Facebook, LINE, Twitter, etc.)? | | | | | | | | | |
