# Supplementary material for: Effect of a Brief Web-Based Educational Intervention on Willingness to Consider Human Papillomavirus Vaccination for Children in Japan: Randomized Controlled Trial
Source: J Med Internet Res. 2021 Sep 27;23(9):e28355. doi: 10.2196/28355 (PMC8506261; doi:10.2196/28355)
Supplement: Multimedia Appendix 3 [file jmir_v23i9e28355_app3.pdf]

CONSORT-EHEALTH Checklist V1.6.2 Report	Manuscript Number	28355
**(based on CONSORT-EHEALTH V1.6), available at [http://tinyurl.com/consort-ehealth-v1-6].**		
**Date completed**		
3/9/2021 2:37:30		
**by**		
Yukio Suzuki		
Effect of a Brief Web-Based Educational Intervention on Willingness to Receive the Human Papillomavirus Vaccine in Japan: A Randomized, Controlled Trial		
TITLE		
**1a-i) Identify the mode of delivery in the title**		
Effect of a Brief "Web-Based" Educational Intervention on Willingness to Receive the Human Papillomavirus Vaccine in Japan: A Randomized, Controlled Trial		
**1a-ii) Non-web-based components or important co-interventions in title**		
**1a-iii) Primary condition or target group in the title**		
The target was not specific disease.		
ABSTRACT		
**1b-i) Key features/functionalities/components of the intervention and comparator in the METHODS section of the ABSTRACT**		
a brief educational intervention using scientific information presented in an easy-to-read format		
**1b-ii) Level of human involvement in the METHODS section of the ABSTRACT**		
We recruited 1,660 participants aged 20 years and older in March 2018 via a webpage and provided them "fully automated" with a 10-item questionnaire related to the following aspects: awareness regarding HPV infection and vaccine, willingness for immunization, and actions for prevention.		
**1b-iii) Open vs. closed, web-based (self-assessment) vs. face-to-face assessments in the METHODS section of the ABSTRACT**		
We recruited 1,660 participants aged 20 years and older in March 2018 via a webpage and provided them fully automated		
**1b-iv) RESULTS section in abstract must contain use data**		
We recruited 1,660 participants.<br>Although only 21.2% of the respondents displayed a willingness toward immunization for their daughters, an additional 4.8% of the respondents in the intervention group reported affirmatively (adjusted odds ratio [aOR]=1.32, 95% CI 1.04-1.69).		
**1b-v) CONCLUSIONS/DISCUSSION in abstract for negative trials**		
INTRODUCTION		
**2a-i) Problem and the type of system/solution**		
This study aimed to assess the effects of these behavioral insights utilizing fundamental scientific information on vaccine benefits, along with statistics on cervical cancer.		
**2a-ii) Scientific background, rationale: What is known about the (type of) system**		
Intervention used in this study is not a system but just a easy-to-read format.		
**Does your paper address CONSORT subitem 2b?**		
Vaccine awareness programs are necessary and campaigns through the media and social network services can play significant roles. In an information-overloaded society, people frequently take decisions related to health issues based on limited information. Therefore, it is critical to consider the influence of behavioral insights to promote change [12]. This broadly refers to concrete approaches based on the knowledge of behavioral science and economics. The Easy, Attractive, Social and Timely (EAST) principles are a simple way of applying behavioral insights to interventions and have been used to change human awareness and behavior [12]. This study aimed to assess the effects of these behavioral insights utilizing fundamental scientific information on vaccine benefits, along with statistics on cervical cancer.		
METHODS		
**3a) CONSORT: Description of trial design (such as parallel, factorial) including allocation ratio**		
We randomly assigned each participant to respond to an identical questionnaire after (Intervention group) or prior to (Control group) providing behavioral insights material (BI-material) using fundamental scientific information presented in an easy-to-read format (as displayed in Figures 1 and 2).<br><br>Participants were randomly allocated (1:1) to each group. Intervention was performed using an automatic web-based allocation system stratified with sex (female/women, male/men) and age (20s, 30s, 40s, 50s, and 60s) of the participants. Randomization was performed by Macromill, Inc web-research system.		
**3b) CONSORT: Important changes to methods after trial commencement (such as eligibility criteria), with reasons**		
No change.		
**3b-i) Bug fixes, Downtimes, Content Changes**		
**4a) CONSORT: Eligibility criteria for participants**		
These were registered members of the research panel owned by Macromill, Inc. (Tokyo, Japan). The participants were 20 years old or above as on March 12th-13th, 2018.		
**4a-i) Computer / Internet literacy**		
**4a-ii) Open vs. closed, web-based vs. face-to-face assessments:**		
We recruited a total of 1,660 participants in March 2018 via a specially designed webpage for this study. These were registered members of the research panel owned by Macromill, Inc. (Tokyo, Japan).<br>Our study was obviously closed.		
**4a-iii) Information giving during recruitment**		
Participants were randomly allocated (1:1) to each group after providing their consent.		
**4b) CONSORT: Settings and locations where the data were collected**		
We recruited a total of 1,660 participants in March 2018 via a specially designed webpage for this study. These were registered members of the research panel owned by Macromill, Inc. (Tokyo, Japan).		
**4b-i) Report if outcomes were (self-)assessed through online questionnaires**		
Our study is performed as web-based trial.		
**4b-ii) Report how institutional affiliations are displayed**		
**5) CONSORT: Describe the interventions for each group with sufficient details to allow replication, including how and when they were actually administered**		
**5-i) Mention names, credential, affiliations of the developers, sponsors, and owners**		
These were registered members of the research panel owned by Macromill, Inc. (Tokyo, Japan).		
**5-ii) Describe the history/development process**		
I described the process in the discussion session about behavioral insights.<br>"Informational materials are designed primarily to increase effectiveness; they are based on a specific purpose rather than a template."		
**5-iii) Revisions and updating**		
This intervention material is the first edition.		
**5-iv) Quality assurance methods**		
Our intervention material is designed primarily to increase effectiveness; they are based on a specific purpose rather than a template.<br><br>The intervention method (BI-material) was designed by us, resulting in a moderate cost; therefore, this intervention is more reliable and cost-effective than those used in previous studies. The first author of this article (YS) is a core member of the behavioral design team in Yokohama, which was established as the first non-governmental nudge unit in Japan. Thus, this BI-material is reliable in view of the BI methodology.		
**5-v) Ensure replicability by publishing the source code, and/or providing screenshots/screen-capture video, and/or providing flowcharts of the algorithms used**		
We displayed the source and flowchart in the manuscript.		
**5-vi) Digital preservation**		
This material is freely used as a material for the education in our department. However we have not provided yet in free access website.		
**5-vii) Access**		
Participants were registered members of the research panel owned by Macromill, Inc. (Tokyo, Japan).		
**5-viii) Mode of delivery, features/functionalities/components of the intervention and comparator, and the theoretical framework**		

Participants were registered members of the research panel owned by Macromill, Inc. (Tokyo, Japan). If this manuscript would be published, we will provide this material through our department website and through SNS.		
**5-ix) Describe use parameters**		
Not applicable for our study.		
**5-x) Clarify the level of human involvement**		
This material was provided through web-site. There were no human involvement.		
**5-xi) Report any prompts/reminders used**		
Not applicable for our study.		
**5-xii) Describe any co-interventions (incl. training/support)**		
Not applicable for our study.<br><br>In the intervention group, we provided the BI-material prior to answering questions related to preventive awareness, following consent for the online study. The control group was provided with the same material as that provided to the intervention group after all responses were completed.<br><br>There are little possibility existing any co-interventions.		
**6a) CONSORT: Completely defined pre-specified primary and secondary outcome measures, including how and when they were assessed**		
The primary outcome is the rate of willingness on the HPV vaccine for their daughters. The secondary outcome is the sub-analysis of sexes.		
**6a-i) Online questionnaires: describe if they were validated for online use and apply CHERRIES items to describe how the questionnaires were designed/deployed**		
Not applicable for our study.		
**6a-ii) Describe whether and how “use” (including intensity of use/dosage) was defined/measured/monitored**		
Not applicable for our study.		
**6a-iii) Describe whether, how, and when qualitative feedback from participants was obtained**		
Not applicable for our study.		
**6b) CONSORT: Any changes to trial outcomes after the trial commenced, with reasons**		
We recruited a total of 1,660 participants in March 2018 via a specially designed webpage for this study. These were registered members of the research panel owned by Macromill, Inc. (Tokyo, Japan).		
**7a) CONSORT: How sample size was determined**		
**7a-i) Describe whether and how expected attrition was taken into account when calculating the sample size**		
The sample size was calculated as powered 80% to detect a 10% effect in the intervention group (increased from 40% in the control to 50% in the intervention group) with a two-sided p value of 0.05. P values less than 0.05 were regarded as significant. The hypothetical baseline willingness rate in the control group was determined based on our previous study. The sample size was calculated as 776 when the effect of intervention estimated a 10% increase. The number of participants recruited was double of the calculated sample size because of the difficulty of estimating the baseline willingness and the intervention effect.		
**7b) CONSORT: When applicable, explanation of any interim analyses and stopping guidelines**		
The primary outcome is the rate of willingness on the HPV vaccine for their daughters. The secondary outcome is the sub-analysis of sexes.		
**8a) CONSORT: Method used to generate the random allocation sequence**		
Participants were randomly allocated (1:1) to each group after providing their consent. Intervention was performed using an automatic web-based allocation system stratified with sex (female/women, male/men) and age (20s, 30s, 40s, 50s, and 60s) of the participants. Randomization was performed by Macromill, Inc web-research system. Participants and investigators were blinded to the distribution (double-blinded). Once the upper limit of each stratum was reached, new participants could not be added to the web system. This ensured uniform distribution of the stratification factors. In the intervention group, we provided the BI-material prior to answering questions related to preventive awareness, following consent for the online study. The control group was provided with the same material as that provided to the intervention group after all responses were completed.		
**8b) CONSORT: Type of randomisation; details of any restriction (such as blocking and block size)**		
Participants were randomly allocated (1:1) to each group after providing their consent. Intervention was performed using an automatic web-based allocation system stratified with sex (female/women, male/men) and age (20s, 30s, 40s, 50s, and 60s) of the participants. Randomization was performed by Macromill, Inc web-research system. Participants and investigators were blinded to the distribution (double-blinded). Once the upper limit of each stratum was reached, new participants could not be added to the web system. This ensured uniform distribution of the stratification factors. In the intervention group, we provided the BI-material prior to answering questions related to preventive awareness, following consent for the online study. The control group was provided with the same material as that provided to the intervention group after all responses were completed.		
**9) CONSORT: Mechanism used to implement the random allocation sequence (such as sequentially numbered containers), describing any steps taken to conceal the sequence until interventions were assigned**		
Not applicable for our study.		
**10) CONSORT: Who generated the random allocation sequence, who enrolled participants, and who assigned participants to interventions**		
Randomization was performed by Macromill, Inc web-research system.		
**11a) CONSORT: Blinding - If done, who was blinded after assignment to interventions (for example, participants, care providers, those assessing outcomes) and how**		
**11a-i) Specify who was blinded, and who wasn’t**		
Participants and investigators were blinded to the distribution (double-blinded).		
**11a-ii) Discuss e.g., whether participants knew which intervention was the “intervention of interest” and which one was the “comparator”**		
Not applicable for our study.		
**11b) CONSORT: If relevant, description of the similarity of interventions**		
Not applicable for our study.		
**12a) CONSORT: Statistical methods used to compare groups for primary and secondary outcomes**		
Statistical evaluation comprised the student’s t-test, the chi-square ($\chi 2$) test, and multiple regression analyses.		
**12a-i) Imputation techniques to deal with attrition / missing values**		
Not applicable for our study. There were no missing values.		
**12b) CONSORT: Methods for additional analyses, such as subgroup analyses and adjusted analyses**		
Same as the primary outcome analysis.		
RESULTS		
**13a) CONSORT: For each group, the numbers of participants who were randomly assigned, received intended treatment, and were analysed for the primary outcome**		
Not applicable for our study. All participants receive intended treatment.		
**13b) CONSORT: For each group, losses and exclusions after randomisation, together with reasons**		
Not applicable for our study.		
**13b-i) Attrition diagram**		
Not applicable for our study.		
**14a) CONSORT: Dates defining the periods of recruitment and follow-up**		
From March 12th to 13th 2018, 1,660 participants were recruited.<br><br>We have no follow-up period.		
**14a-i) Indicate if critical “secular events” fell into the study period**		
Not applicable for our study.		
**14b) CONSORT: Why the trial ended or was stopped (early)**		
Not applicable for our study.		
**15) CONSORT: A table showing baseline demographic and clinical characteristics for each group**		
The following variables displayed no significant differences between the two groups: marital status (P=.96), children (P=.84), household income (P=.58), sexual experience (P=.26), education (P=.44), medical background (P=.50), and tobacco use (P=.64) (Table 1).		
**15-i) Report demographics associated with digital divide issues**		
Not applicable for our study.		
**16a) CONSORT: For each group, number of participants (denominator) included in each analysis and whether the analysis was by original assigned groups**		
**16-i) Report multiple “denominators” and provide definitions**		
Not applicable for our study. All participants answered questionnaire and received intervention as allocated.		
**16-ii) Primary analysis should be intent-to-treat**		
Not applicable for our study. All participants answered questionnaire and received intervention as allocated.		
**17a) CONSORT: For each primary and secondary outcome, results for each group, and the estimated effect size and its precision (such as 95% confidence interval)**		

Only 21.2% of the respondents displayed a favorable attitude toward HPV immunization for their daughters (Q6). However, an additional 40 (4.8%) participants responded affirmatively in the intervention group (aOR=1.32, 95% CI=1.04-1.69) compared to those in the control group (Table 2). For Q7, there were additional 33 (3.9%) satisfied respondents willing for their sons' vaccination in the intervention group (aOR=1.38, 95% CI=1.05-1.80) compared to those in the control group (Table 2).		
**17a-i) Presentation of process outcomes such as metrics of use and intensity of use**		
Not applicable for our study.		
**17b) CONSORT: For binary outcomes, presentation of both absolute and relative effect sizes is recommended**		
Only 21.2% of the respondents displayed a favorable attitude toward HPV immunization for their daughters (Q6). However, an additional 40 (4.8%) participants responded affirmatively in the intervention group (aOR=1.32, 95% CI=1.04-1.69) compared to those in the control group (Table 2). For Q7, there were additional 33 (3.9%) satisfied respondents willing for their sons' vaccination in the intervention group (aOR=1.38, 95% CI=1.05-1.80) compared to those in the control group (Table 2).		
**18) CONSORT: Results of any other analyses performed, including subgroup analyses and adjusted analyses, distinguishing pre-specified from exploratory**		
Differences were identified in Q6 (Men; aOR=1.46, 95% CI=1.05-2.02 vs Women; aOR=1.20, 95% CI=0.83-1.73) and Q7 (Men; aOR=1.53, 95% CI=1.08-2.18 vs Women; aOR=1.21, 95% CI=0.80-1.83). The awareness in men showed a significant positive change (by 8.2%, P=0.02, Supplement 1) upon intervention by fundamental scientific information; however, this was not observed in women (P=0.22, Table 3, Supplement 2).<br><br>There were no additional analysis other than planned analysis.		
**18-i) Subgroup analysis of comparing only users**		
Not applicable for our study.		
**19) CONSORT: All important harms or unintended effects in each group**		
Not applicable for our study.		
**19-i) Include privacy breaches, technical problems**		
Not applicable for our study.		
**19-ii) Include qualitative feedback from participants or observations from staff/researchers**		
Not applicable for our study.		
DISCUSSION		
**20) CONSORT: Trial limitations, addressing sources of potential bias, imprecision, multiplicity of analyses**		
**20-i) Typical limitations in ehealth trials**		
Third, selection bias existed as the respondents were Japanese enrolled by an internet survey company.		
**21) CONSORT: Generalisability (external validity, applicability) of the trial findings**		
**21-i) Generalizability to other populations**		
Our study reveals a positive outlook towards the HPV vaccination following brief, web-based educational intervention, especially among male participants. Such an approach is extremely effective to overcome challenges related to communication and information overload.		
**21-ii) Discuss if there were elements in the RCT that would be different in a routine application setting**		
Not applicable for our study.		
**22) CONSORT: Interpretation consistent with results, balancing benefits and harms, and considering other relevant evidence**		
**22-i) Restate study questions and summarize the answers suggested by the data, starting with primary outcomes and process outcomes (use)**		
We conducted a web-based randomized controlled trial (RCT) to assess the benefits of BI-material employing fundamental scientific information and motivate individuals to take the HPV vaccine. Our results showed that providing brief scientific information could increase the positive awareness of HPV vaccination. This effect was observed typically among male participants. Similar minor interventions may potentially modify mindsets favorably. However, such brief digital information failed to affect the mindset in women. In terms of the coronavirus disease (COVID-19) pandemic, a difference in awareness of prevention strategies was observed between the sexes; therefore, it is essential to build a method appropriate for sex subgroups to transform general behavior via the internet and social networking service (SNS).		
**22-ii) Highlight unanswered new questions, suggest future research**		
We recognize several limitations in this study. First, the sustainability of effective change was not evaluated. Typically, with respect to health issues, taking action requires time. Therefore, a study should assess not only a change in mindset but also the appropriate course of action. We have already performed a RCT (UMIN000039273) assessing the sustainability of general acceptance and concrete behavior for HPV vaccination.		
Other information		
**23) CONSORT: Registration number and name of trial registry**		
UMIN000049745<br>URL; https://www.umin.ac.jp/english/		
**24) CONSORT: Where the full trial protocol can be accessed, if available**		
Not applicable for our study.		
**25) CONSORT: Sources of funding and other support (such as supply of drugs), role of funders**		
We received research funding from the Japan Agency for Medical Research and Development (grant no. 15ck0106103h0102).		
**X26-i) Comment on ethics committee approval**		
The study protocol was approved by the Institutional Research Ethics Committee of Yokohama City University School of Medicine (A180200004). The trial registration number is UMIN000049745.		
**x26-ii) Outline informed consent procedures**		
In the intervention group, we provided the BI-material prior to answering questions related to preventive awareness, following consent for the online study. The control group was provided with the same material as that provided to the intervention group after all responses were completed.		
**X26-iii) Safety and security procedures**		
Not applicable for our study.		
**X27-i) State the relation of the study team towards the system being evaluated**		
We have no COI between study team and the survey company.		
